# Role of tumor microenvironment in cancer promotion, development of drug resistance and cancer treatment

**DOI:** 10.1186/s43046-025-00317-8

**Published:** 2025-09-15

**Authors:** Duaa e Fathah, Samina Ejaz

**Affiliations:** The Islamia University of Bahawalpur, Bahawalpur, Pakistan

**Keywords:** Tumor microenvironment, Cancer, Angiogenesis, CAR-T cell therapy, PD-1/PD-L1

## Abstract

Cancer is a multifactorial disease and the second leading cause of death worldwide after cardiovascular disease. Initially, it was considered a genetic disease or gene expression disorder, but now it is regarded as a tumor microenvironment (TME) disease. The TME consists of cancer cells, endothelial cells, fibroblasts, and immune cells that interact with each other. These interactions support tumor growth by providing nutrients via altered metabolic mechanisms such as glutamine metabolism, aerobic glycolysis, and fatty acid metabolism. The by-products of these altered metabolic pathways interfere with the function of surrounding cells and thus lead to cancer progression. The role of metabolic crosstalk highlights the intricate relationship between the cancer cells and their TME. This review comprehensively analyzes recent studies to enhance understanding of the metabolic crosstalk in TME. It highlights how tumor-associated macrophages and fibroblasts reprogram lipid and glucose metabolism to create an immunosuppressive environment. This review also provides information about the role of hypoxia-induced HIF-1α signaling in the promotion of lactate accumulation. This factor in turn ensures tumor cells’ survival and makes them resistant to anti-cancer drugs. Further, we have discussed therapeutic approaches targeting TME, including use of PD-1, PD-L1 inhibitors, CAR-T cell therapy, and oncolytic viruses to improve patient outcomes. Besides this, clinical studies involving the estimation of lactate, GLUT1, and HIF-1α levels may help to recognize high-risk patients and develop guidance for personalized metabolism-targeting therapies. In the long run, such studies can ultimately improve patient outcomes and thus reduce disease burden.

## Introduction

Cancer is the second leading cause of mortality after cardiovascular disease worldwide [1]. In 2024, reported cancer cases in the USA were 2 million along with 611,720 deaths [2]. In Pakistan, the total burden of cancer cases in 2022 was 185,748, and the mortality rate was 118,631 [3]. Cancer is a multifactorial disease, and one of its major causative factors is the altered genetic makeup. In patients, cancer progression is facilitated by mutant proteins that regulate the cell cycle, apoptosis, and DNA repair processes [4]. Treatment of cancer has always been a challenging task for clinicians. Although conventional treatment methods, like chemotherapy, surgery, and radiotherapy, are in common practice, recent advancements in medical research and technology have provided clinicians with more effective therapeutic tools including targeted therapy, stem cell therapy, and ablation therapy. Besides this, nanoparticles are widely used as a delivery system across various advanced cancer therapies including immune therapy and stem cell therapy. They help enhance the targeting and bioavailability of therapeutic agents such as hyaluronic acid and natural compounds. Natural antioxidants such as curcumin, berberine, and quercetin offer anti-inflammatory and pro-apoptotic effects, help to overcome oxidative stress, and inhibit tumor growth [5–7].

Numerous new technologies are currently in clinical trials; some have already been approved by the FDA. Among these methods, surgery is helpful at an early stage of the disease progression. In contrast, radiation and chemotherapy can affect the normal functioning of cells, tissues, and organs. In treatment with chemotherapy, drug resistance is a severe issue in which cancer cells develop a resistance to the administered drug [8]. Tumor heterogeneity is considered the leading cause of chemoresistance as tumor heterogeneity exhibits a diverse nature of cancer cells within and between tumors. This means that cancer cells can vary from each other in their morphology, their metabolic activities, and gene expression patterns. So, in the treatment of solid cancers like breast cancer (BC), tumor heterogeneity leads to complications because variations are evident even in the patient’s tumors suffering from the same type of cancer, and one patient’s tumor is different from another patient’s tumor. Thus, tumor heterogeneity complicates cancer treatment [9].

The tumor microenvironment (TME) is known to support chemoresistance and carcinogenesis, and thus targeting TME can be a potential therapeutic approach in cancer [10]. The TME is comprised of tumor cells, surrounding stromal cells such as endothelial and stromal fibroblasts, and immune cells such as macrophages, lymphocytes, and the non-cellular components of extracellular matrix (ECM), including fibronectin, collagen, laminin, and hyaluronan. The key players of TME are the tumor cells which promote carcinogenesis by communicating via complex signaling such as notch, VEGF-VEGFR2, and JAK/STAT with non-cancerous cells to facilitate cancer metastasis and development of resistance to treatment [11]. Among the immune cells in the TME, macrophages play a crucial role in tumor metastasis. Initially, macrophages in the primary tumor support tumor growth when these cancer cells are preparing to metastasize to other body tissues. The resident macrophages in the distal tissues create a welcoming environment called a pre-metastatic niche upon receiving signaling messages generated by primary tumor-derived extracellular vesicles that could alter the behavior of these macrophages. Eventually, cancer cells settle in these tissues and recruit other immune cells, such as monocytes, that become tumor-associated macrophages and support tumor metastasis. Thus, in primary tumors, macrophages aid in tumor cells growth and prepare metastatic sites for the metastasis and development of secondary tumors [12]. During the metastasis process, stromal cells present in TME play a role in both suppression and metastasis. However, the entire function of stromal cells is valuable for cancer cell’s persistence and spread. The stromal cells such as mesenchymal stem cell/s (MSCs), lymphocytes, pericytes, fibroblasts, and endothelial cells contribute to tumor progression by promoting ECM remodeling, drug resistance, angiogenesis, and metastasis [13]. It is therefore suggested that TME is not just a static observer, but it actively encourages cancer progression [14].

The primary goal of this review is to analyze the complexity of TME to understand how metabolic crosstalk in TME promotes cancer metastasis. We have discussed the role of some TME-targeting therapeutic approaches available worldwide. Some of these therapies are under clinical trials, but further research is recommended to develop more TME-based therapeutic tools to achieve the goal of optimal patients’ outcomes during treatment.

## The function of tumor microenvironment (TME) in cancer progression

### Complexity of tumor microenvironment (TME)

A compromised immune system and alterations in homeostatic regulatory mechanisms lead to the emergence of tumors. The interactions among cancerous and immune cells influence the proliferation of tumor cells in the TME. The tumor cells’ survival is ensured, and the spread of cancer cells to distant tissues during metastasis is facilitated due to the interaction of cellular and structural constituents of TME [15]. Among the cellular constituents of the TME, various types of cells such as immune cells, endothelial cells, and extracellular matrix are present, which collectively participate in tumor progression. Cancer stem cells (CSCs) act as a determining factor that devotes intrinsic heterogeneity to malignant tumors [16].

The relationship between cancer, mast cells, and inflammation is conflicting, as it has a dual role in both cancer progression and protection. The mast cells surround certain tumors, especially in mammary adenocarcinoma. The tumor cells can release certain chemicals or chemo-attractants that recruit mast cells; in turn, these mast cells release factors such as histamine, VEGF (vascular endothelial growth factor), IL-8, and heparin that consequently promote angiogenesis and metastasis [17, 18]. However, in breast cancer (BC) different outcomes were noticed in the case where IL-4 secreted by the mast cells induced BC cells’ apoptosis and thus reduced the tumor growth [19]. Emerging evidence recommends that innate immune cells, ncluding neutrophils, which are inflammatory mediators during the early stage of cancer, become immunosuppressive when the tumor progresses. In TME, tumor-associated neutrophils (TANs) can exhibit two phenotypes, N1 (tumor-suppressive), and N2 (tumor-promoting) depending on the tumor stage. On the other hand, macrophages perform diverse functions in the immune response to cancer cells. These macrophages are categorized into two subtypes, including M1 and M2 macrophages. These subtypes were observed to be activated by different stimuli. For instance, M1 macrophages are activated by interferon-gamma (IFNγ) and LPS (lipopolysaccharides), enabling them to eradicate tumor cells by killing them. Conversely, M2 macrophages are activated by IL-4, IL-13, or IL-10 and are associated with cancer progression [20].

Other immune cells that are involved in the cancer-associated immune response include natural killer cells(NKs) and myeloid-derived suppressor cells (MDCSc). These NKs engage in destroying tumor cells, but their ability in TME is reduced as tumor cells escape from destruction by NKs with the aid of different mechanisms. Such mechanisms occur in a way that these tumor cells coat themselves in collagen to trigger inhibitory NK receptors and additionally use platelets as a shield to avoid detection by NKs. Moreover, MDSCs (myeloid-derives suppressor cells) contribute to immunosuppression by suppressing T cells. Additionally, these cells promote tumors by enhancing tumor cell stemness and stimulate angiogenesis and metastasis by IL-6 secretion, which promotes EMT [21].

The immune response against cancer is mediated by both the innate and adaptive immune systems. Specifically, in the adaptive immune system, the cytotoxic CD8 + T cells (CTLs) play an important role in tumor prevention and eradication. In many cancer cases, the CTLs-mediated tumor fails to clear cancer cells, resulting in disease progression in various cancer patients, largely because of CTLA4 (Cytotoxic T-lymphocyte-associated protein 4) exhaustion. This exhaustion is stimulated by prolonged exposure to tumor cell antigens and is characterized by elevated expression of surface markers, including CTLA4 and PD-1 (programmed cell death protein 1). Consequently, exhausted CD8 + T cells release pro-inflammatory cytokines, tumor growth factor beta (TGF-β), and interferon-gamma (IFN- γ), and suppress the cancer cells’ killing ability of CTLs. Recently, CTLA-4 and PD-1 have been recognized as drug targets for immunotherapy-based cancer treatments in patients with various cancers such as melanoma. The combined use of anti-PDL1 and anti-CTLA-4 is considered a first-line treatment following clinical trials [22].

The fibroblasts are another crucial component of TME that are spindle-shaped cells of connective tissue. These cells perform key functions in the tumor stroma, including depositing extra-cellular matrix(ECM), promoting wound healing, and modulating inflammation. In TME, the fibroblasts can exhibit an altered phenotype that is labeled by elevated expression of certain markers such as fibroblast activation protein (FAP), α-smooth muscle actin (α-SMA), and increased ECM protein secretion such as type I collagen and fibronectin. In TME, the fibroblasts activated via interactions with cancer cells called cancer-associated fibroblasts (CAFs) can encourage metastasis by facilitating underlying mechanisms including invasion and colonization of cancer cells at secondary sites. Among the stromal cells, CAFs have become key players due to their abundance in various solid tumors such as BC, CRC (colorectal cancer), and pancreatic cancer. Interestingly, the CAFs are involved in both cancer progression and suppression. Moreover, these CAFs can communicate with tumor cells and such interactions take place via paracrine signals including metabolites, cytokines, exosomes, and diverse functions of ECM [23]. The interactions between cancer cells and CAFs can also occur via different mechanisms including tumor-promotive CAF secretions that contain cytokines and growth factors involved in cancer cell proliferation, survival, and resistance to cancer therapy.

Additionally, CAFs promote inflammation, ECM stiffness, and tumor angiogenesis by releasing matrix metalloproteinases (MMPs) and cytokines [24]. The TME is responsible for immune system resistance in cancer cells due to factors like hypoxia, nutrient deficiency, and acidity, which alter the immune cells’ metabolism and their function. Both tumor and immune cells need an enormous amount of nutrients, including amino acids, glucose, and fatty acids, to fulfill their energy needs. This leads to competition between immune and cancer cells, which leads to metabolic adaptations in various metabolic pathways such as fatty acid synthesis, aerobic and anaerobic glycolysis, glutaminolysis, tryptophan catabolism, oxidative phosphorylation, and reprogramming of immune cells. This reprogramming affects immune cells such as natural killer cells, tumor-associated macrophages, T cells, and dendritic cells and thus alters their response in the TME. Apart from these, some host factors, including obesity, caloric deficit, gender, microbiota, infection, and smoking, might be contributory to immune modulation, immune checkpoint inhibition, and antitumor immunity [25]. Some TME molecules that are observed to play a role in cancer progression are discussed in Table 1.

**Table 1:** Summary of tumor microenvironment molecules identified in cancers

Sr. no	Molecule type	Nature	Examples	High expression in TME	Low expression in TME	References
1	Cytokines	Non-enzymatic protein	Interleukins (IL-1, IL-6, IL-10, TNF-A)	Lungs, prostrate, breast, CRC cancer	Unknown	[26–29]
2	Cadherin adhesion molecules (CAMs)	Non-enzymatic protein	VCAM-1, ICAM-1, E-selectins	Breast pelvic cancers	E-cadherin (breast cancer)	[30–32]
3	Other inflammatory mediators	Lipid, peptide, molecules (include various types of molecules)	Prostaglandins, Bradykinin	Colorectal, breast, gastric, lung, glioblastoma	Unknown	[33–36]
4	Enzymes	Protein	MMP2/9, Cyclooxygenase 1–2, tyrosine kinase	Ovarian, bladder, oral, retinoblast, colorectal, triple-negative breast cancers	Unknown	[37–40]
5	Growth factors	Cytokine (protein)	TGF-β, VEGF	Hepatocellular carcinoma, ovarian	Unknown	[41–43]
6	Growth factor receptors	Protein	EGFR, VEGFR	Breast cancer	Unknown	[44–46]

Hypoxic microenvironment is also a hallmark of solid tumors and is documented in 90% of them. Measuring the hypoxic state is challenging due to variations in tumor size and the limitation of the measurement method. Moreover, tissue oxygenation is a crucial aspect, as oxygen content can differ significantly between various tissues within the same organ. In a hypoxic microenvironment, cancer cells can survive due to the activation of HIF-α (hypoxia-inducible factor alpha) which belongs to the HIF (hypoxia-inducible factor) family. This family comprises two distinct subunits α (HIF-1α and HIF-2α, and HIF-3α) and β (HIF-1β). Hypoxia promotes carcinogenesis by regulating underlying mechanisms such as inducing DNA strand breaks including SSB (single strand break) and DSB (double-strand break), and impairs DNA repair pathways such as MMR (mismatch repair) and HR (homologous recombination). The up-regulation of HIF-α is due to an abnormality in various signaling pathways including Notch, P13K, mTOR, JAK-STAT3, NF-KB, MAPK, Wnt/β-catenin. Moreover, the deletion of TSGs (tumor suppressor genes) including P53 and PTEN also contribute to the elevated levels of HIF-α. The accumulation of HIF-1α is due to the lost function of PHVL (Prolyl Hydroxylase Von Hippel-Lindau Inhibitor) under hypoxic conditions. The PHVL is also known as non-Hippel-Lindau protein. The main function of PHVL is to direct proteasomal degradation of HIF-1α and HIF-2α in physiological conditions. The accumulated HIF-1α dimerizes with HIF-1β, and enters the nucleus to bind with HRE (hypoxia response elements) in promoter regions in DNA, and regulates transcription of downstream target genes, and ultimately consequences in cancer progression [47].

Thus, immunosuppression, hypoxia, and chronic inflammation are the three characteristic features of TME that effectively coordinate various steps of tumor development, such as immune escape, drug resistance, and metastasis [15].

### Metabolic crosstalk in TME promotes tumor growth

The survival and proliferation of cancer cells, processes of high metabolic demand, create a nutrient-deficient, hypoxic, and acidic tumor microenvironment. The acidity in TME is the key factor in modulating the immune response that affects the metabolism of immune cells. The metabolic reprogramming of immune cells, especially myeloid cells including dendritic cells, myeloid-derived suppressor cells (MDSC), and tumor-associated macrophages (TAMs) results in cancer progression by promoting immunosuppression and angiogenesis [48]. Cancer cells, due to metabolic flexibility, can adjust from a nutrient-deprived environment to a lactate and glutamine-rich environment, where these cancer cells can utilize glutamine and lactate as energy sources. In TME, the interaction between CAFs and cell-ECM regulates the metabolic switch, and CAFs release metabolites such as aspartate, pyruvate, and lactate, which are known to support the growth of different cancer cell types [49].

Most of the cells use glucose as an energy source, which is metabolized through glycolysis, a multi-step process, to yield pyruvate. In normal conditions, pyruvate gets access to mitochondria and is oxidized to oxygen and carbon dioxide by the TCA cycle to produce ATP. In cancer cells, instead of complete oxidation, pyruvate is converted to lactate by lactate dehydrogenase enzyme. The lactic acid production in the presence of oxygen is called the Warburg effect. It is a process by which cancer cells prefer glycolysis over oxidative phosphorylation for energy, even when no oxygen is available [50]. Thus, cancer cells acquire metabolic variations in the absence of essential nutrients, to meet their energy needs and to maintain their survival [51]. The lactate produced in cancer cells promotes tumor growth, angiogenesis, and immune tolerance by activating lactate receptor GPR81, which is expressed in both tumor and non-tumor cells such as macrophages and dendritic cells. So, GPR81 is a possible target for cancer therapy [52]. Although tumor cells’ need for mitochondrial ATP production decreases, their requirement for biosynthetic precursors and demand for molecule NADPH increase to support their cellular processes and growth. To compensate and maintain a functional TCA cycle, cancer cells may depend on elevated glutaminolysis, which is a process in which transporters, SLC7A5 and SLC1A5, glutamine are transported into the cells and transformed to glutamate and further to alpha-ketoglutarate (α-KG) by many enzymes, including glutaminase (GLS) and glutamate dehydrogenase (GDH) to make ATP production possible via TCA cycle and to provide carbon skeleton, nitrogen, and sulfur for macromolecule synthesis that are necessary for cancer cells growth. Glutamine is highly needed in tumor cells and also in rapidly dividing cells such as lymphocytes. Furthermore, the activity of mitochondrial enzymes involved in glutamine/glutamate oxidation is high in cancer cells in contrast to normal cells. Glutaminolysis promotes tumor growth in two ways: either by encouraging cell proliferation or by inhibiting apoptosis. The leading role of glutaminolysis in cell growth is to provide intermediary metabolites in the TCA cycle. For instance, glutaminolysis generates α-KG (alpha-ketoglutarate), thereby replenishing the TCA cycle and contributing to energy production. Glutamine is essential for the biosynthesis of nucleotides, hexosamine, and some nonessential amino acids.

Moreover, glutaminolysis is vital in regulating biological processes including mTOR signaling, autophagy, redox balance, and apoptosis [53, 54]. Cell multiplication requires lipids for new biological membrane synthesis. Fatty acids (FAs) are the building blocks obtained from endogenously synthesis and via exogenously (diet). Transporters including CD36, LDLR, FABPs (fatty acid binding proteins), and FATPs (fatty acid transport proteins) facilitate the uptake of FAs from blood circulation. FA synthesis is vital for tumorigenesis as cancer cell/s activate new fatty acid synthesis pathways in contrast to normal cells that rely on circulating FAs. FA synthesis utilizes citrate as a carbon source for the synthesis of FAs.This citrate is produced by the TCA cycle in normal cells.

**Fig. 1:**
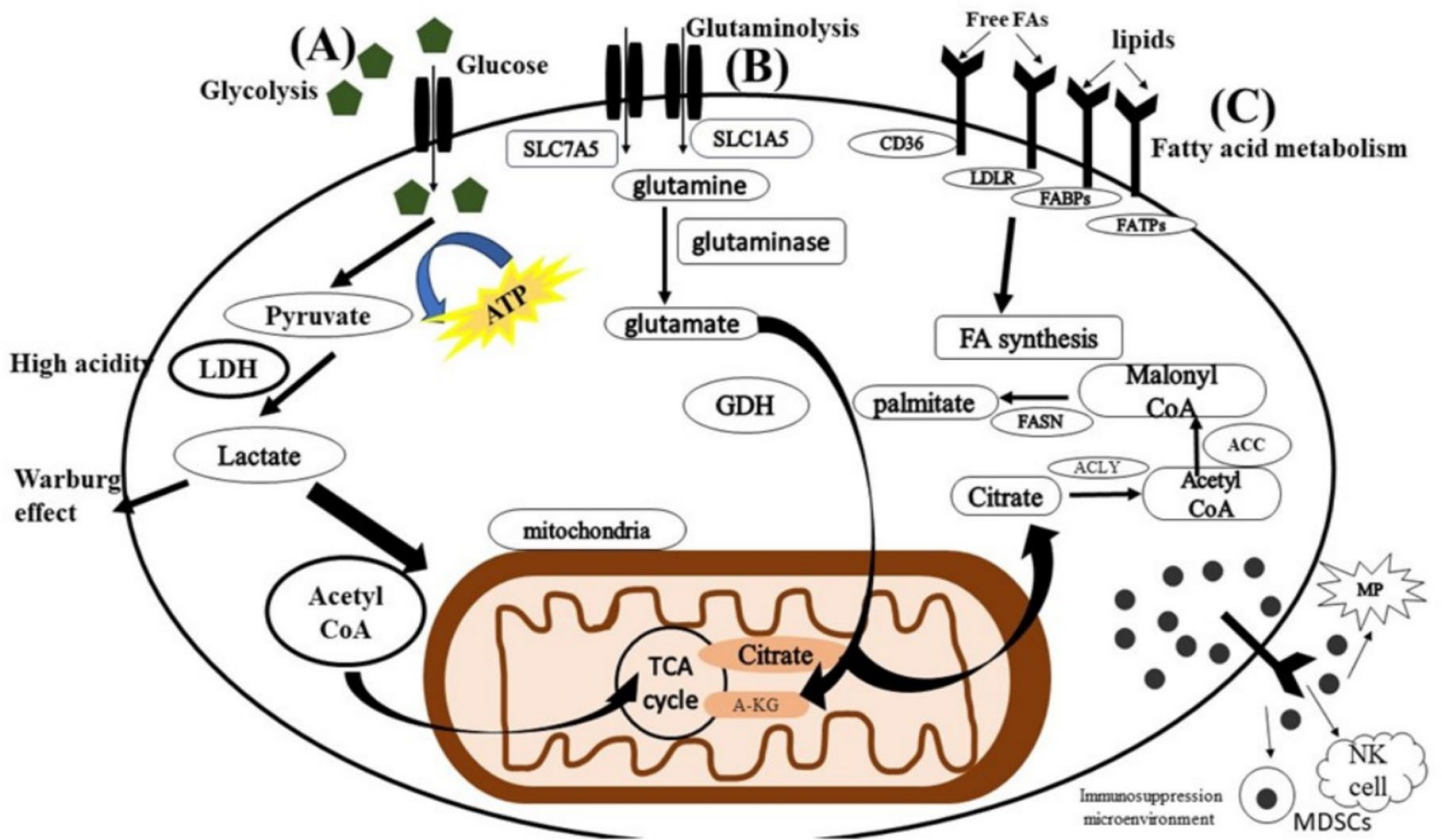
Metabolic crosstalk in cancer cells. Cancer cells show altered metabolism including Warburg effect, glutaminolysis, and elevated fatty acid synthesis. These pathways are interconnected and support tumor growth. The fatty acid synthesis produces lipids for the membrane formation of cells, and glutaminolysis provides intermediates to the TCA cycle, which in turn generates energy for cell survival. LDH (lactate dehydrogenase), GDH (glutamate dehydrogenase), FASN (fatty acid synthase), ACLY (ATP citrate lyase), ACC (acetyl CoA carboxylase), MP (macrophages), MDSCs (myeloid derives suppressor cells), and NK (natural killer cells)

In contrast, during hypoxia, and in cancer cells with malfunctioning mitochondria where the TCA cycle is slowed down, citrate is produced by reductive carboxylation of glutamine-derived α-ketoglutarate by the NADPH-dependent isocitrate dehydrogenase. Further, this citrate is split into oxaloacetate and acetyl-CoA via ATP-citrate lyase (ACLY). Next, acetyl-CoA is transformed to malonyl-CoA via acetyl-CoA carboxylase (ACC), leading to FA synthesis and intricate FA elongation via FASN (fatty acid synthase) until palmitate is produced. Additional changes of FAs can be performed by enzymes including desaturases and elongases at distinct carbon lengths. In the glycerol phosphate pathway, free FAs are converted into a form known as an ester. In this process, glycerol combines with esters to form glycerol phosphate esters, the building blocks of sphingolipids and phospholipids that, along with cholesterol, form important constituents of all biological membranes. For energy storage, FAs can also be stored as cholesterol esters and triacylglycerols (TAGs). When cells need energy, they can be generated via Faβ-oxidation (FAo). Recent studies showed that in cancer progression, cancer cells scavenge circulating FAs from their surroundings as well as rely on their fatty acid synthesis pathway, and scientists did not realize for a long time that exogenous FAs also promote tumor growth [55, 56]. Alteration in lipid metabolism affects the immune response by influencing fatty acid synthesis in cancer cells. This leads to increased fatty acid storage in the TME. This stored fat is utilized by regulatory T cells (Treg cells), resulting in immunosuppression against tumors or leading to tumor growth [57]. The information has been summarized in Fig. 1.

## Therapeutic approaches targeting TME

### Immune-based therapies

In recent years, significant progress has been achieved in cancer treatment. Immune checkpoint inhibitors (ICIs) and CAR-T cell therapy are the immunotherapies that strengthen the immune system to fight against cancer cells. Immunotherapy, in combination with conventional treatment including radiation and chemotherapy or immunotherapy alone, has attained significant achievements in the treatment of several cases of cancer. The CTLA-4 (cytotoxic T-lymphocyte antigen) and PD-1 (programmed cell death protein 1) are the co-inhibitory T cell surface receptors. Their primary function is to reduce the activity of hyperactive T cells, thereby protecting healthy cells from an excessive immune response. These receptor proteins act as checkpoints, and cancer cells utilize these checkpoints to evade the immune response against them, thus leading to immune tolerance [58]. As of now, the FDA has approved some PD-1 inhibitors including cemiplimab, nivolumab, and pembrolizumab, and PD-L1 inhibitors are avelumab, durvalumab, and atezolizumab. The first and only CTLA-4 inhibitor that is approved by the FDA is Ipilimumab [59]. Some FDA-approved PD-L1 and CTLA-4 inhibitors are shown in Table 2.

**Table 2:** Some FDA-approved checkpoint inhibitors

Sr. no	Drugs	Types	Drug ID	Class of compound	Molecular formula	Cancer Type	FDA approved	Year of approval	Target
1	Sotorasib	PD-1 inhibitor	PubChem 137,278,711	Acrylamide	C30H30F2N6O3	Non-small cell lung cancer	Yes	2021	KRAS
2	Avelumab	PD-L1 inhibitor	DB11945	Monoclonal antibodies	C6374H9898N1694O2010S44	Merkel cell carcinoma, metastatic urothelial carcinoma, or renal cell carcinoma	Yes	2017	Inhibits PD-1 activation
3	Dostarliab	PD-1 inhibitor	483,928,936	Monoclonal antibodies	C6420H9832N1680O2014S44	Endometrial cancer	Yes	2023	PD-1
4	Atezolizumab	PD-L1 inhibitor	DB11595	Monoclonal antibodies	Not reported	Metastatic urothelial carcinoma	Yes	2016	PD-L1
5	Durvalumab	PD-L1 inhibitor	DB11714	G1 kappa monoclonal antibody	C6502H10018N1742O2024S42	Urothelial carcinoma, lung cancer	Yes	2017	BH17
6	Ipilimumab	CTLA-4 inhibitor	DB06186	Monoclonal antibody	C6572H10126N1734O2080S40	Metastatic or unresectable melanoma	Yes	2011	CTLA-4

In the tumor microenvironment (TME), the dendritic cells, which are antigen-presenting cells (APC), process specific tumor peptides (TAA) and complex these peptides with MHC (major histocompatibility complex) molecules. The APCs present these processed antigens to T cells. In the immune synapse, the T cell activation needs two signals. The first signal is mediated by the antigen presentation by APC via MHC-TCR (Major Histocompatibility Complex) (T cell receptor) interactions. The second way of T cell activation depends on a signal that can be either an activator or inhibitor. This signal is controlled by two receptors including T cell-CD28, which binds with CD80/CD86 on APC for T cell activation, and CTLA-4, which competes with T cell-CD28 for the same binding site to inhibit T cells. During the T cell/s activation, intracellular events are facilitated by the programmed cell death 1 receptor (PD-1). The extracellular surface of the T cells harbors a variety of receptors including PD-1, CD28, and TCR/CD3 receptor complex. The receptors are occupied by their specific ligands, such as CD80/CD86, PD-L1, and MHCI and MHCII, respectively, in the activated T cells. This receptor-ligand binding brings all immunological synapses closer to interact with each other. The intracellular tails of CD4 and CD8 interact with Src kinase Lck (P56Lck), which phosphorylates the tyrosine residues on intracellular tails of CD28, PD-1, and also the CD3 chain of the TCR/CD3 complex. The phosphorylation of receptor tyrosine-based switch motif (ITSM) motif on the intracellular tail of PD-1 signals for the recruitment of Src homology region 2 domain-containing phosphatase 2 (SHP-2) leading to the activation of its phosphatase activity. At the same time, the phosphorylated tail of CD28 recruits molecules including Grb 2 and PI-3 K, along with other signaling molecules. However, at the immunological synapse, the cytoplasmic tail of the CD28 receptor can be dephosphorylated by the SHP-2, thereby preventing further downstream signaling. The PD-1 can also be dephosphorylated by SHP-2, leading to the regulation of the PD-1 inhibitory pathway, which is an essential mechanism that prevents excessive immune response. CD-28 is crucial in the T cell activation. The CD28 receptor, present on the T cell, works along with TCR, sends signals, and helps the T cell in its multiplication, survival, and IL-2 production. But if CD28 cannot bind to two other molecules including Grb2 and PI3K, the signaling gets disrupted and makes the T cell unable to perform its effector function. PD-1 remains out of the immune synapse in the absence of its ligand, which is PD-L1. The CD28 has an affinity for binding with CD80 and CD86. But CTLA-4 receptor proteins bind strongly with these CD80 and CD86 receptor proteins and indirectly disrupt the CD28 signaling by reducing the availability of the CD28 binding site. The monoclonal antibodies, like pembrolizumab, which targets PD-1, and atezolizumab, which targets PD-L1, can block the PD-1/PD-L1 inhibitory effects. Moreover, anti-CTLA-4, that is ipilimumab, can allow CD28 to function properly.

**Fig. 2:**
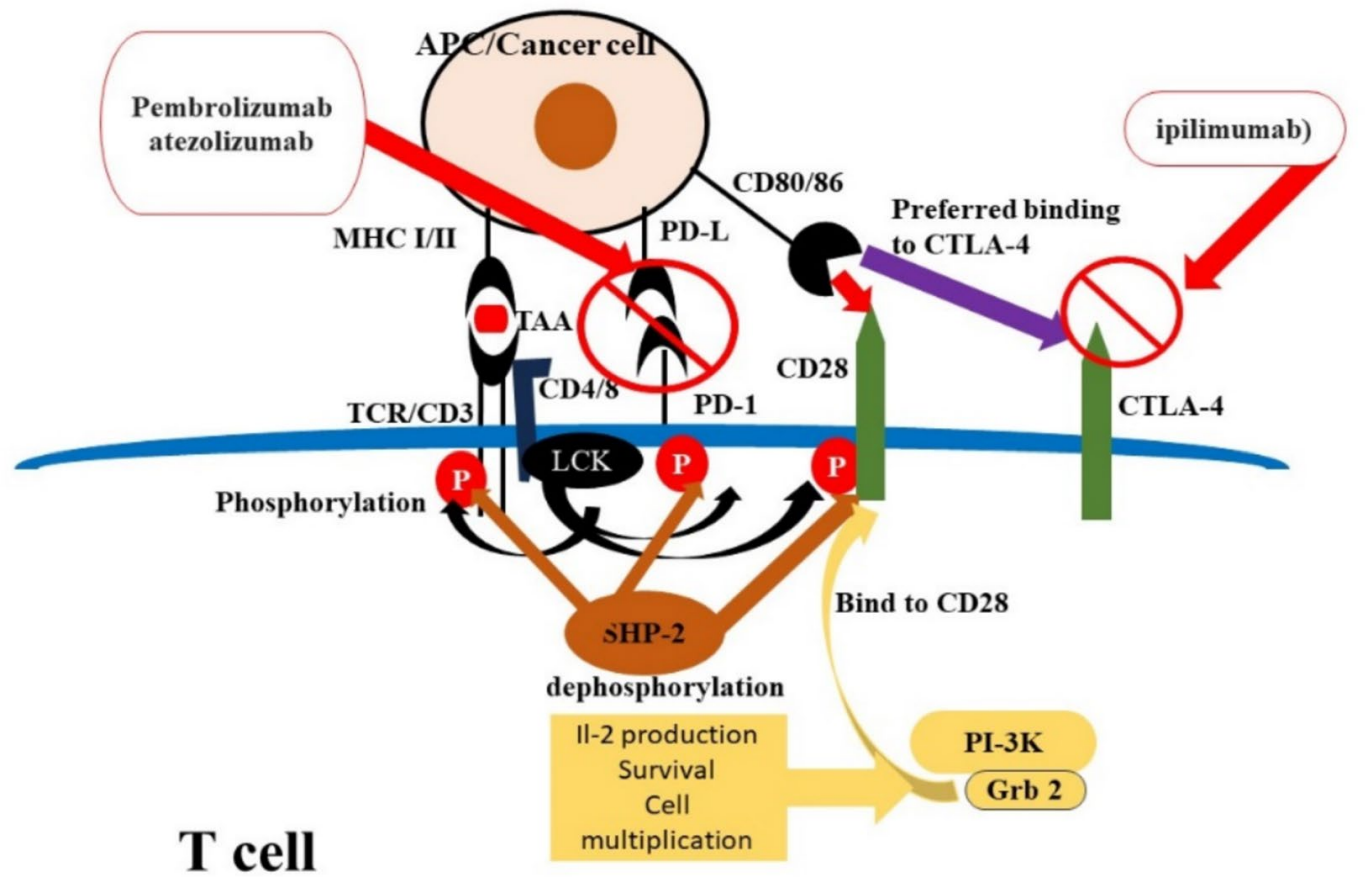
Intracellular signaling during T cell activation. During T cell activation, intracellular events are mediated by programmed cell death (PD-1). When T cells get activated, the extracellular surface CD28, PD-1, and TCR/CD3 complex receptors bind with their specific ligands*,* including PD-L, MHC, CD80, and CD86 on APCs (antigen-presenting cells) in the immune synapse. A protein called Lck associated with the intracellular tails of CD4 and CD8 phosphorylates the cytoplasmic domain of CD28, PD-1, and CD3, initiating further downstream signaling. CTLA-4 is an inhibitory receptor that indirectly disrupts CD28 signaling by binding to CD80 and CD86, making them unavailable for CD28. The main consequence of this process is the precise T cell regulation and its effective immune response

The PD-1 receptor regulates T cell-mediated responses by inhibiting the cytokine secretion, including TNF-α, IL-2, and IFN-γ. Moreover, it also affects T cell multiplication by interfering with the CD28 co-stimulatory signaling pathway. The aberrant expression of PD-1 has been observed in many immune cells within the TME, such as dendritic cells (DC), T cells, B cells, and natural killer cells (NK). The anti-PD-1 inhibitors have been utilized in the treatment of various cancers, such as melanoma, non-small-cell lung cancer (NSCLC), Merkel cell carcinoma, and head and neck squamous cell carcinoma (HNSCC). There are two ligands for PD-1, which are PD-L1 and PD-L2, also known as CD274 and B7-H1, which is expressed by both immune and tumor cell/s, making it a useful biomarker in various cancer patients, as it predicts response to anti-PD-1/PD-L1 antibodies. The primary role of PD-L1 is to inhibit immune response against cancer by binding to the PD-1 receptor, thereby restricting tumor cell killing by inhibiting T cell proliferation and migration. The anti-PD-L1 has been used in the treatment of many cancers, such as melanoma, NSCLC, MCC, and HNSCC. CTLA-4 is expressed solely on T cell, where it primarily inhibits the CD28 function by not binding to it. Despite that, although CTLA-4 does not bind with CD28, it can bind to the CD28 homolog B7 on B cells and APCs, thereby preventing T cells from killing cancer cell/s [60, 61] as shown in Fig. 2.

Another immunotherapy, alongside checkpoint inhibitors, is CAR-T cell therapy, which has been used to treat leukemias, multiple myeloma, and lymphomas [62]. In the treatment of lymphoma patients, anti-PD-1 immunotherapy combined with CAR-T cell therapy has shown potency, but fever is the common side effect [63]. CAR-T cell therapy is considered for cancer immunotherapy due to quick recognition of tumor-specific antigens by T cells, providing a basis for cancer immunotherapy. T cells circulate everywhere in the body and scan for MHC on APC s [44]. In contrast, CAR-T cells can recognize antigens in an MHC-independent manner. Activation of T cell by phosphorylation of receptor tyrosine-based activation motif (ITAM) and increased T cell multiplication release of cytokines, including IL-12, TNF, IL-2,4. The NK cells and macrophages are recruited, and their function is strengthened by Interleukin-12 (IL-12). The activated T cells and CAR-T cell work together, thereby creating cytotoxicity by releasing granzyme and perforins, and creating a Fas/FasL (Fas ligand) based death signal [64].

An adequate amount of blood is drawn from the patient, from which T cells are purified, and then modified in a laboratory. After in vitro activation and amplification, T cells are altered to express TCRs (T cell receptors) and CARs (chimeric antigen receptors) on the T cell surface by viral vector transfection including retroviruses and lentiviruses. The CAR-T (chimeric antigen receptors T cell therapy) cells are injected into the patient's body to improve the anti-tumor response [65]. Despite the advantages of CAR-T cell therapy, there are many disadvantages including antigen escape, poor persistence, and immune rejection of therapeutics. Apart from challenges, CAR-T cell-based immunotherapy remains a novel concept. In treating hematological cancers, additional clinical trials have been conducted to evaluate their effectiveness [66]. Some clinical trials and FDA-approved CAR-T cell therapies have been discussed in Table 3. Many other FDA-approved drugs or vaccines (Table 4) are used to treat various types of cancer, but some have adverse effects as shown in the table.

**Table 3:** FDA-approved CAR-T cell therapies

Sr. no	CAR-T cell product	Target antigen	Indication	FDA date approval	Clinical trials ID	References
1	Tisagenlecleucel	CD19	Pediatric and young adult patients with CD19 + relapsed or refractory B cell ALL	2017	NCT02435849 (2015–05-06)	[67]
2	Anti-GD2 T cells	Disialoganglioside (GD2)	Relapsed or Refractory Neuroblastoma	Not reported	NCT02761915 (2016–05-04)	[68]
3	Axicabtagene ciloleucel	CD19	Refractory large B cell lymphoma	2017	NCT02348216 (2015–01-28)	[69]
4	Anti-CD19	CD19	Pediatric/young adult patients with relapsed or refractory B cell ALL	Not reported	NCT03373071 (2017–12-14)	–
5	KTE-X19	CD19	Relapsed or refractory Mantle-cell lymphoma	2020	NCT02601313 (2015–11-10)	[70]
6	Idecabtagene vicleucel	BCMA	Relapsed and refractory multiple myeloma	2021	NCT03361748 (2017–12-05)	[71]
7	Lisocabtagene maraleucel	CD19	Relapsed or refractory large B cell lymphomas	2021	NCT02631044 (2015–12-15)	[72]

*NCT* national clinical trial (https://www.clinicaltrials.gov/)

**Table 4:** FDA-approved drugs and vaccines for various cancers

Sr. no	Drug/vaccine	Targets	Indications	Side effects	References
1	Imatinib	ABL-BCR	CML (chronic myeloid leukemia)	T135I mutation	[73, 74]
2	Trastuzumab	HER2	Breast cancer	Cardiac dysfunction in combination with anthracycline	[75]
3	Rituximab	CD20	B cell malignancies	Unknown	[76]
4	Pembrolizumab/Nivolumab	PD-1	Head and neck squamous cell carcinoma (HNSCC)	Unknown	[77]
5	Ipilimumab	CTLA-4	Melanoma	Immune-related adverse effect	[78, 79]
6	Bevacizumab	VEGF	Colorectal cancer	Unknown	[80]
7	Paclitaxel	Microtubules	Triple-negative breast cancer	Unknown	[81]
8	Tamoxifen	Estrogen receptor	Positive breast cancer	Unknown	[82]
9	Gardasil/Ceravix	HPV (humanpapilloma virus)	Cervical cancer, HNSCC	Unknown	[83]

As immunotherapy is a promising approach to treat cancer, it does not work for many patients. Primary resistance refers to patients who do not respond to immunotherapy at all, and acquired resistance refers to patients who respond first but then stop responding over time. The mechanisms behind these resistances include alterations in TME, loss of antigen presentation, and upregulation of alternative immune checkpoints. To improve the outcomes, clinicians have combined immunotherapy with other treatment modalities such as chemotherapy and radiation [84]. For instance, in lung cancer (NSCLC) combining pembrolizumab with chemotherapy drugs like platinum and pemetrexed improves survival [85]. While in kidney cancer, combinations like avelumab with pembrolizumab and axitinib respond better than sunitinib alone [86]. In other cancers, these combinations also worked; in TNBC, pembrolizumab with chemotherapy enhanced the survival rate [87] while radiotherapy combined with pembrolizumab showed a better response in lung cancer than immunotherapy alone. However, these combinations can cause side effects such as liver toxicity with vemurafenib and ipilimumab [86]. In contrast, CAR-T cell therapy has been proven to be a successful approach in hematological malignancies, but it has shown limited effectiveness in solid tumors [88].

### Targeting angiogenesis within TME

**Fig. 3:**
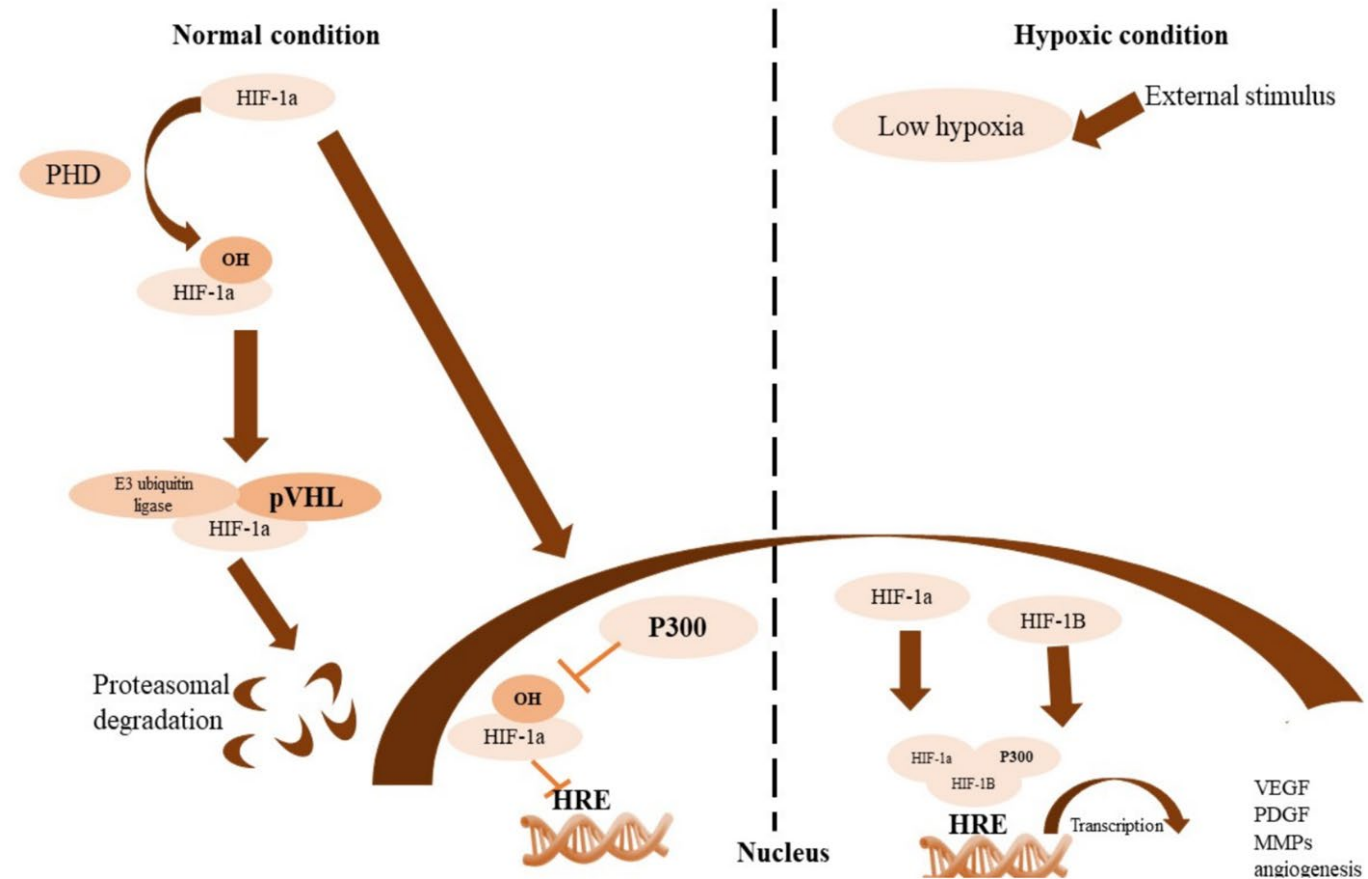
Angiogenesis signaling pathway in normal and hypoxic conditions: HIF-1α is hydroxylated by PHD enzymes, leading to proteasomal degradation via pVHL, but in hypoxic conditions, low oxygen levels reduce the activity of PHD enzymes; HIF-1α stabilizes, permitting it to enter the nucleus and bind to HRE (hypoxia response elements) on DNA, leading to the transcription of target genes such as VEGF and PDGF and promoting angiogenesis

Angiogenesis is a complex process involved in the progression of many solid tumors such as breast cancer (BC), colorectal cancer (CRC), and renal cell carcinoma (RCC). This process is accomplished by the elevated production of growth factors (GFs) including platelet growth factor (PGF), vascular endothelial growth factor (VEGF), endothelial growth factor (EGF), and fibroblast growth factor 2 (FGF2). Hypoxia is a hallmark of TME, which is closely related to tumor angiogenesis, progression, recurrence, and also drug resistance. The hypoxia-inducible factor1 (HIF-1) is a heterodimeric transcription factor that regulates cell adaptation to hypoxia, erythropoiesis, and energy metabolism and is involved in cell survival. HIF-1 is composed of two subunits: HIF-1α, an oxygen regulator, which becomes active when oxygen levels are low, and HIF-1β, a nuclear protein. Under normal conditions, HIF-1α is degraded due to hydroxylation of its proline residues by proline hydroxylase domain (PHD) enzymes, which enables E3 ubiquitin ligase and pVHL (von Hippel Lindau protein) to mediate its ubiquitination and subsequent proteasomal degradation. Besides, hydroxylation of asparagine residues in HIF-1α regulates its transcriptional activity by disrupting its interaction with co-activation factor p300, thereby inhibiting VEGF expression and angiogenesis. However, under hypoxic conditions, reduced oxygen concentration decreases hydroxylation, stabilizing HIF-1α and facilitating its nuclear translocation. HIF-1α then forms a complex with HIF-1β, which interacts with co-activator p300 and binds with the hypoxia response element (HRE) located on the HIF target genes (Fig. 3). This interaction leads to transcriptional activation of downstream target genes encoding MMPs (matrix metalloproteins), PDGF (platelet-derived growth factor), and VEGF (vascular endothelial growth factor). This complex process encourages tumor angiogenesis by inhibiting apoptosis in the epithelial cells and promotes tumor cell survival [89, 90].

The most extensively used anti-angiogenic therapy is inhibition of VEGFA/VEGRR2 binding. The first FDA-approved anti-VEGFA monoclonal antibody, Bevacizumab, is used to treat various cancers including cervical, colorectal, and renal cell cancers. Many of the ongoing clinical trials have focused on the combined effect of chemotherapy and anti-VEGF therapy, but many other trials have reported no improvement in cancer patients. For example [91], a study aimed to analyze the long-term effect of combined Bevacizumab and chemotherapy for the treatment of early triple-negative breast cancer (TNBC) but showed no significant improvement in overall survival (OS) of early TNBC (triple-negative breast cancer) patients [92]. In contrast, the combined effect of Bevacizumab and chemotherapy has improved OS in the first-line treatment of advanced non-small cell lung cancer. The FDA-approved drugs for metastatic tumors are axitinib, sunitinib, everolimus, lenalidomide, Bevacizumab, ramucirumab, pazopanib, regorafenib, cabozantinib, lenvatinib, sorafenib, Ziv-aflibercept, vandetanib, and thalidomide [93]. Some of these drugs are discussed in Table 5, and the information of clinical trials of anti-VEGF alone or in combination with other drugs is discussed in Table 6.

**Table 5:** FDA-approved drugs treating angiogenesis for metastatic tumors

Sr. no	Drugs	Targets	Cancer type	Chemical formulas	Structures	Status	Drug ID
1	Axitinib	Vascular endothelial growth factor receptor (VEGFR) and kinase inhibitor	Advanced renal cell carcinoma	C_22_H_18_N_4_OS		Approved	DB06626
2	Sunitinib	Multi-targeted receptor tyrosine kinase (RTK) inhibitor	Renal cell carcinoma, imatinib-resistant gastrointestinal stromal tumor	C_22_H_27_FN_4_O_2_		Approved	DB01268
3	Everolimus	(mTOR) kinase inhibitor	HER2-negative breast cancer, progressive neuroendocrine tumors of pancreatic origin	C_53_H_83_NO_14_		Approved	DB01590
4	Pazopanib	Protein tyrosine kinase inhibitor	Renal cell cancer and advanced soft tissue sarcoma	C_21_H_23_N_7_O_2_S		Approved	DB06589
5	Regorafenib	Kinase inhibitor	Metastatic colorectal cancer, metastatic gastrointestinal stromal tumors, and hepatocellular carcinoma	C_21_H_15_ClF_4_N_4_O_3_		Approved	DB08896
6	Sorafenib	Multi kinase inhibitor	Hepatocellular carcinoma, renal carcinoma, and differentiated thyroid carcinoma	C_21_H_16_ClF_3_N_4_O_3_		Approved	DB00398
7	Vandetanib	kinase inhibitor	Metastatic medullary thyroid cancer	C_22_H_24_BrFN_4_O_2_		Approved	DB05294

**Table 6:** Clinical trials based on anti-VEGF alone and in conjunction with other approaches

Sr. no	Cancers	Medications	Study phase	Study duration	Enrollment	Study location	Status	Clinical trials ID
1	Gastrointestinal Stromal tumor	RAD001, Imatinib 600 mg/day, Imatinib 800 mg/day	I and II	2002–2008	117	Worldwide	Completed	NCT01275222 (2011–01-12)
2	Gastrointestinal Stromal tumor, Brain and solid tumor	Peginterferon-alpha 2b (PegIFNa2b); Imatinib	II	2007–2009	8	USA	Completed	NCT00585221 (2008–01-03)
3	Advanced refractory cancer	Imatinib	II	2014–2022	14	Korea	Active, not recruiting	NCT02461849 (2015–06-03)
4	Breast cancer	Standard chemotherapy, bevacizumab [Avastin], trastuzumab [Herceptin]	II	2008–2014	52	France	Completed	NCT00717405 (2008–07-17)
5	Ovary cancer	Bevacizumab, Laboratory Biomarker Analysis	II	2008–2013	36	International	Completed	NCT00748657 (2008–09-08)
6	Solid tumor	Bevacizumab, Dasatinib	I	2008–2018	50	USA	Completed	NCT01445509 (2011–10-03)
7	Hepatocellular Carcinoma	Regorafenib, Nivolumab	I/II	2020–2024	69	Spain	Active, not recruiting	NCT04170556 (2019–11-20)
8	Gastric, Esopheagal, melanoma, cervical	Ipilimumab, Pembrolizumab, Nivolumab, Regorafenib, Carboplatin, Paclitaxel, FOLFIRI Protocol, Bevacizumab	I/II	2023–2026	134	USA	Recruiting	NCT06047379 (2023–09-21)
9	Metastatic colorectal, pancreatic cancer	Olaptesed pegol–Monotherapy, Olaptesed pegol + Pembrolizumab–Combination Therapy	I/II	2017–2020	20	Germany	Completed	NCT03168139 (2017–05-30)
10	Glioblastoma	Olaptesed pegol, Radiotherapy, Bevacizumab, Pembrolizumab	I/II	2019–2024	27	Germany	Active, not recruiting	NCT04121455 (2019–10-10)
11	Multiple myeloma	IMNOVID		2014–2022	775	International	Completed	NCT02164955 (2014–06-17)
12	Multiple myeloma	Pomalidomide	II	2009–2015	84	France	Completed	NCT01053949 **(**2010–01-22)
13	Metastatic pancreatic cancer	Galunisertib, Durvalumab	I	2016–2019	37	International	Completed	NCT02734160 **(**2016–04-12)
14	Hepatocellular carcinoma	LY2157299, Sorafenib, Placebo	II	2014–2021	132	International	Completed	NCT02178358 **(**2018–06-08)
15	Colorectal, Hodgkin, rectum cancer	Vactosertib, Fludarabine Phosphate, Cyclophosphamide IL-2, natural killer cells	I	2022–2024	12	USA	Suspended	NCT05499122

(https://www.clinicaltrials.gov/)

Apart from growth factors, some genes were found to be dysregulated. For instance, the deregulation of the HOX (homeobox-containing gene) gene impacts pathways that can affect cancer cells proliferation, cancer cells survival, and motility, and these disrupted events have consequences in the formation of aggressive tumors. The deregulation of the HOX gene induces the upregulation of angiogenic factors that signal myeloid cell recruitment. Among the HOX cluster genes, HOXB9 is a transcription factor. Researchers have identified a protein, HOXB9, that plays a vital role in the development of solid cancers, including breast, colorectal, and endometrial. Although it is not the primary cause of cancer, HOXB9 contributes to cancer spread by promoting cell motility and angiogenesis. So, HOXB9 can be targeted for anti-angiogenic treatment and used as a possible prognostic biomarker for those patients who benefited from anti-angiogenic therapies [94].

The EGF (epidermal growth factor) is another growth factor involved in angiogenesis. It helps endothelial cells to proliferate, which line the blood vessels. The EGF sends signals through its receptor EGFR (epidermal growth factor receptor). These signals activate downstream signaling pathways, including MAPK, P13K/AKT/PKB, PLC/PKC, and STAT. It has been observed by researchers that HIF-α enhances the EGF/EGFR signaling, which in turn helps HIF-α to work better, allowing cancer cells to survive under hypoxic conditions, leading to angiogenesis and tumor progression. The high expression of EGFR is usually found in BC, bladder, pancreatic, and non-small cell lung cancer. In the early treatment of lung cancer, mutations in the EGFR *T790M* gene are the primary cause of drug resistance to EGFR kinase inhibitors, including erlotinib and gefitinib, as reported by many studies. Although angiogenesis is the cause of cancer metastasis, with an in-depth understanding of TME, angiogenesis, and drug resistance, these issues may be resolved in the future [89, 95].

The sensitivity of anti-angiogenic therapy varies based on tumor types, and some cancers show positive responses such as hepatocellular, renal carcinoma, neuroendocrine, thyroid, ovarian, and cervical cancers; but in breast, colorectal, glioblastoma, prostate, and pancreatic cancers, only partial effectiveness or no responses were observed [96–101]. Apart from the benefits of anti-angiogenic drugs on patient survival, resistance to these drugs often occurs during clinical treatment, leading to treatment failure and poor consequences. Increasing evidence shows that the interaction between bone marrow-derived cells, stromal cells, and tumor cells enables tumors to escape anti-angiogenic therapy. Currently, drug resistance has become the main factor that interrupts the efficiency of anti-angiogenic therapy [102]. Anti-angiogenic therapy works differently in contrast to chemotherapy, in which cancer cells develop resistance due to genetic mutations affecting the drug’s target. In anti-angiogenic therapy, tumor cells can find alternative ways to survive using various unstated mechanisms, when VEGF signaling is blocked by anti-angiogenic drugs [103].

### Stromal cell targeting strategies (CAF targeted therapies)

**Fig. 4:**
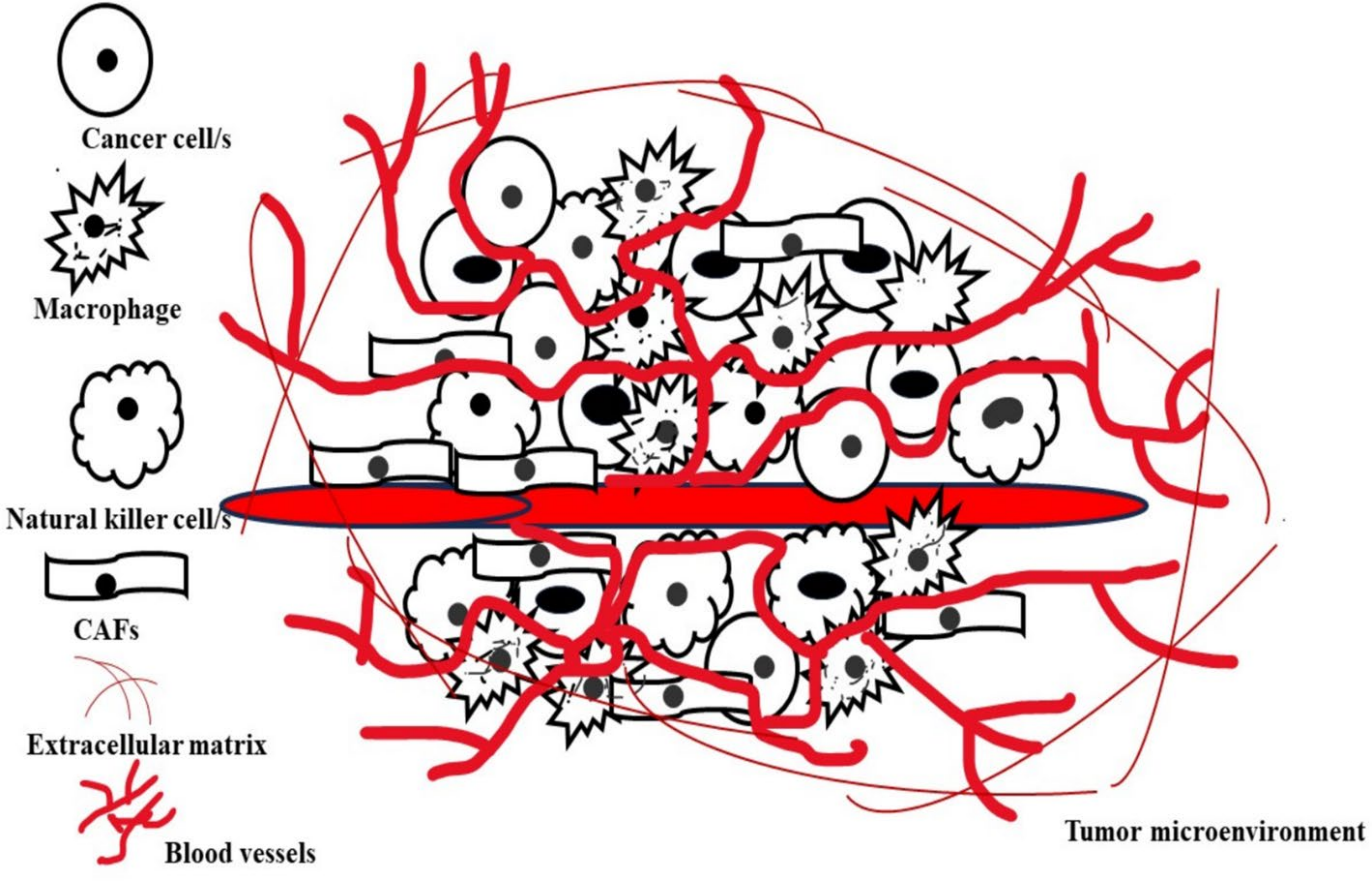
Cells of tumor microenvironment (TME). A TME surrounds a tumor, comprising various cell types that interact with each other. The components of TME are stromal, immune, and cancer cells as well as extracellular matrix components

Stromal cells are a diverse group of connective tissue cells, which provide structural support to tissues and organs. These stromal cells are of various types, each with a different shape and function, such as fibroblasts, melanocytes, and pericytes, which are found throughout the body, as well as the stem cells including adipose tissue-derived stem/stromal cells (ASCs) and bone-marrow-derived mesenchymal stem/stromal cells (MSCs). Some specialized stromal cells are unique to certain organs, including fibroblastic reticular cells in lymphoid tissues and interstitial cells of Cajal in the gastrointestinal tract (GIT) [104]. The TME is composed of diverse molecular and cellular components, and stromal cells are the essential cellular component of TME, which facilitate cancer metastasis, induce drug resistance, and immune escape. Stromal cells are produced by two sources and contribute to the complexity of cancer development. When a tumor grows, it can send a signal to nearby non-cancerous stromal cells. These nearby stromal cells can then move toward the tumor and become part of its support system. The second source of stromal cells is transdifferentiation, in which stromal cells change into different types of stromal cells to support the tumor (Fig. 4).

However, with a deep understanding of stromal cells, researchers have observed the dual role of stromal cells in both progression and inhibition of cancer. The mesenchymal stem cells (MSCs), another major class of stromal cells, have different roles in each stage of tumor development. In the early stage of tumors, exogenous MSCs have an inhibitory effect. These cells can reduce cancer cell proliferation and induce apoptosis. Moreover, MSCs can also prevent tumor cells from entering the S phase of the cell cycle.

Additionally, MSCs in TME can promote tumors via many mechanisms including cell-to-cell contact, enhancement of angiogenesis, secretion of biomolecules, inhibition of immune cell activity, and its conversion to CAFs [105]. Along with that, different stromal cells, such as myofibroblasts, cancer-associated mesenchymal stem cells, and fibroblasts create a complex communication network with tumor-infiltrating immune cells to inhibit the anti-tumor response. This communication network keeps immune cells away from the tumor site and makes it harder for the immune cells to defend against the tumor [106].

Cancer-associated fibroblasts (CAFs) are the vital components of the microenvironment surrounding the TNBC (triple negative breast cancer). Within the stroma, CAFs show aberrant gene expression patterns, which provide valuable insights into the prognosis of TNBC. The upregulated pathways related to chemokine signaling recruit the immune cells via chemokine signals, contributing to inflammation and ECM remodeling, which is the alteration of the extracellular matrix. All these irregular events lead to tumor growth. In the TNBC condition, these upregulated pathways in CAFs create a microenvironment hospitable to tumor progression [107]. Some of the drugs targeting the stromal cells are mentioned in Table 7..

**Table 7:** Information on clinical trials using drugs that target stromal cells in different cancers in combination with other strategies

**Sr. no**	**Cancers**	**Medications**	**Study phase**	**Study duration**	**Enrollment**	**Study location**	**Status**	**Clinical trials ID**
1	Gastrointestinal Stromal tumor	RAD001,Imatinib 600 mg/day, Imatinib 800 mg/da	I and II	2002-8	117	Worldwide	Completed	NCT01275222 (2011-01-12)
2	Gastrointestinal Stromal tumor, Brain and solid tumor	Peginterferon-alpha 2b (PegIFNa2b); Imatinib	II	2007-9	8	United state	Completed	NCT00585221 (2008-01-03)
3	Advanced refractory cancer	Imatinib	II	2014-22	14	Korea	Active, not recruiting	NCT02461849 (2015-06-03)
4	Breast cancer	standard chemotherapy, bevacizumab [Avastin], trastuzumab [Herceptin]	II	2008-14	52	France	Completed	NCT00717405 (2008-07-17)
5	Ovary cancer	Bevacizumab, Laboratory Biomarker Analysis	II	2008-13	36	International	Completed	NCT00748657 (2008-09-08)
6	Solid tumor	Bevacizumab, Dasatinib	I	2008-18	50	United state	Completed	NCT01445509 (2011-10-03)
7	Hepatocellular Carcinoma	Regorafenib, Nivolumab	I/II	2020-24	69	Spain	Active, not recruiting	NCT04170556 (2019-11-20)
8	Gastric, Esopheagal, melanoma, cervical	Ipilimumab, Pembrolizumab, Nivolumab, Regorafenib, Carboplatin, Paclitaxel, FOLFIRI Protocol, Bevacizumab	I/II	2023-26	134	United state	Recruiting	NCT06047379 M(2023-09-21)
9	Metastatic colorectal, pancreatic cancer	Olaptesed pegol – Monotherapy, Olaptesed pegol + Pembrolizumab - Combination Therapy	I/II	2017-20	20	Germany	Completed	NCT03168139 M(2017-05-30)
10	Glioblastoma	Olaptesed pegol, Radiotherapy, Bevacizumab, Pembrolizumab	I/II	2019-24	27	Germany	Active, not recruiting	NCT04121455 (2019-10-10)
11	Multiple myeloma	IMNOVID		2014-22	775	International	Completed	NCT02164955 (2014-06-17)
12	Multiple myeloma	Pomalidomide	II	2009-15	84	France	Completed	NCT01053949 (2010-01-22)
13	Metastatic pancreatic cancer	Galunisertib, Durvalumab	I	2016-19	37	International	Completed	NCT02734160 (2016-04-12)
14	Hepatocellular carcinoma	LY2157299, Sorafenib, Placebo	II	2014-21	132	International	Completed	NCT02178358 (2018-06-08)
15	Colorectal, Hodgkin, rectum cancer	Vactosertib, Fludarabine Phosphate, Cyclophosphamide IL-2, Natural Killer Cells	I	2022-24	12	United States	Suspended	NCT05499122

(https://www.clinicaltrials.gov/)

Targeting cancer-associated fibroblasts (CAFs) is a therapeutic strategy that is used for the treatment of various cancers. CAF-targeted therapies show better responses in cancers like colorectal, breast, and thyroid cancers, making these cancers more favorable targets for such approaches. In contrast, pancreatic, melanoma, and sarcoma showed lower efficacy in several clinical trials [108–110]. The challenges that are faced include heterogeneity in their function, both anti- and pro-tumorigenic CAFs, and the absence of specific biomarkers for CAFs. However, by exploring the role of CAFs in drug resistance, scientists have suggested that combining CAF-targeting drugs with other therapies, including immunotherapy and chemotherapy, will improve patient outcomes [111].

### Oncolytic virus therapy for TME

To treat solid tumors including breast cancer (BC), radiotherapy was used in 1895, and had satisfactory outcomes; till 1900, surgery was the main monotherapy due to severe tissue damage. Chemotherapy was first used to treat a solid tumor in 1958, but in the 1950 s and 1960 s, scientists were concerned with treating cancer via oncolytic viruses. The first oncolytic virus was discovered in 2005 [112]. The Oncorine (H101) was the first commercialized oncolytic virus in the world, synthesized by the Shanghai Sunway, and was approved by the Chinese SFDA in November 2005 for nasopharyngeal carcinoma [113]. The oncolytic viruses are a new category of therapeutic agents containing anti-tumor activity which work via dual mechanisms of action. One way is the initiation of systematic anti-tumor immune response, and the second is selective oncolysis, i.e., tumor cells killing [114].

Oncolytic viruses are genetically modified, targeting or killing specific tumor cells. These oncolytic viruses trigger a systematic immunity by directly targeting the tumor cells or leading to tumor cell lysis. After infection in the tumor cell, the virus starts replicating, producing viral proteins; inducing an oxidative stress response that encourages the activation of pathways associated with autophagy mechanisms. Oncolysis activates the release of tumor-associated antigens (TAAs), which are presented to naïve T cells in the lymph nodes by antigen-presenting cells (APC) [115]. In addition to immunogenic cell death (ICD), the oncolytic viruses can modify the TME, making it more susceptible to immune attack and less hospitable to cancer cells [116]. In contrast to cancer cells, various pathways get activated in normal cells to clear viral particles. These include interferons (IFN) and toll-like receptors that detect pathogen-associated molecular patterns (PAMPS). The TLR’s signaling activates downstream factors such as TRAF3, IRF3, IRF7, RIG-1, and the JAK/STAT pathway. In cancer cell/s, IRF3, IRF7, and RIG-1 are down-regulated, which lowers the detection chance of viral particles by RIG-1 and TLRs, making cancer cells more susceptible to viral replication, eventually leading to oncolysis [114].

Oncolytic therapy is used in the treatment of various cancers, including melanoma, nasopharyngeal, bladder, pancreatic, colorectal, and lung cancers [117]. Even though oncolytic viruses are the most potent therapeutic agents for cancer treatment, due to the heterogeneity of cancer cells, a single type of oncolytic virus is not adequate to kill all cancer cells, as some cancer cells show resistance. Moreover, the most challenging part of oncolytic virus therapy is to recognize the delivery method that fits perfectly with the patient’s immune system and identify the virus that activates the immune response against cancer cells. At present, many therapeutic viruses such as herpes simplex virus (HSV), vaccinia virus, measles virus, adenovirus, coxsackievirus, and reovirus are undergoing clinical trials for the treatment of cancer. To achieve more reliable results, oncolytic virus therapy should be used in combination with immunotherapy and chemotherapy [118].

The information related to some clinical trials of virotherapy is summarized in Table 8.

**Table 8:** Clinical trials of virotherapy

**Sr. no**	**Virotherapy**	**Cancers**	**Study phase**	**Enrollment**	**Study location**	**Study duration**	**Status**	**Clinical trial ID**
1	LOAd703(Adenovirus)	OvarianColorectal cancers,Pancreaticadenocarcinoma	I/II	46	Sweden	2018-24	Active, not recruiting	NCT03225989(2017-07-21)
2	R130(simplex virus type I)	Sarcoma, carcinoma,Breastandpancreatic cancer	I	20	China	2023-26	Recruiting	NCT05860374(2023-05-16)
3	Hv01(vaccinia virus)	Advanced solid tumors	I	24	China	2023-25	Recruiting	NCT05914376(2023-06-22)
4	*Pelareorep(reovirus)	Recurrent plasma cell myeloma	I	23	Unitedstates	2018-22	Completed	NCT03605719(2018-07-30)
5	A21(coxsackievirus)	Melanoma	I	9	Australia	2007-9	Completed	NCT00438009(2007-02-21)

(https://www.clinicaltrials.gov/).*wild type Reovirus (pelareorep) along with Carfilzomib, Dexamethasone, Nivolumab were used for myeloma treatment. Talimogene laherparepvec (T-VEC) is the first FDA-approved modified oncolytic herpes simplex virus for melanoma treatment [65]

## Conclusion

This review highlights that tumor microenvironment (TME) is highly complex and can vary from patient to patient, which makes it difficult to target. As TME is a potential target, it is essential to identify TME biomarkers, which can be immune biomarkers like PDL-1, angiogenic biomarkers like VEGF and HIF-1, cancer-associated fibroblasts (CAFs), and stromal cells. Researchers have already developed therapies to target these biomarkers. Although these approaches show significant patient outcomes, the immunosuppressive nature of TME, tumor heterogeneity, and therapy resistance remain a challenge in cancer treatment. Some therapies exhibit limitations; notably, CAR-T cell therapy has limited efficacy in solid tumors’ treatment, while CAF-targeting strategies are hindered by the dual role of fibroblast subtypes. Hence, in this regard, research should focus on the design of multi-functional inhibitors, for instance, that can simultaneously control immune checkpoints and target CAFs. Moreover, biomarker-based patient stratification can identify subgroups of patients who are more likely to benefit from specific TME-targeted treatment. Furthermore, molecular profiling can guide the selection of personalized treatment. Using rational drug design and combination therapies, an improvement in patient outcomes can be achieved.

### Future prospectives

The combination of oncolytic viral therapy with angiogenic therapy can be a potential approach, as this combination can inhibit blood vessel formation and the selective replication of oncolytic viruses can lead to the lysis of cancer cells. Hence, it is suggested that this combination should be considered in clinical trials.

## Limitations

Despite the advancement, targeting TME still faces challenges due to its complexity. Immune checkpoint inhibitors (ICIs) are applicable only in patients, especially where the immune system is involved. Similarly, cancer-associated fibroblasts (CAFs) exhibit heterogeneity in function, comprising both anti- and pro-tumorigenic CAFs. The indiscriminate targeting of both anti- and pro-tumorigenic CAFs could be insufficient, if not deleterious. On the other side, anti-angiogenic therapies (VEGF inhibitors) lose efficiency over time as tumor cells find other ways for angiogenesis. Like platelet-derived growth factor (PDGF) stabilize tumor blood vessel formation by recruiting pericytes, helping them survive even after inhibiting VEGF. Thus, the treatment success in cancer patients is limited due to resistance to existing treatment options and the diverse nature of TME across patients.

## Data Availability

No datasets were generated or analysed during the current study.
